# *FgPex3*, a Peroxisome Biogenesis Factor, Is Involved in Regulating Vegetative Growth, Conidiation, Sexual Development, and Virulence in *Fusarium graminearum*

**DOI:** 10.3389/fmicb.2019.02088

**Published:** 2019-09-20

**Authors:** Xiangjiu Kong, Hao Zhang, Xiaoliang Wang, Theo van der Lee, Cees Waalwijk, Anne van Diepeningen, Balazs Brankovics, Jin Xu, Jingsheng Xu, Wanquan Chen, Jie Feng

**Affiliations:** ^1^State Key Laboratory for Biology of Plant Diseases and Insect Pests, Institute of Plant Protection, Chinese Academy of Agriculture Sciences, Beijing, China; ^2^Biointeractions and Plant Health, Wageningen Plant Research, Wageningen, Netherlands

**Keywords:** *Fusarium graminearum*, peroxisome, *FgPex3*, fungal growth, virulence

## Abstract

Peroxisomes are involved in a wide range of important cellular functions. Here, the role of the peroxisomal membrane protein PEX3 in the plant-pathogen and mycotoxin producer *Fusarium graminearum* was studied using knock-out and complemented strains. To fluorescently label peroxisomes’ punctate structures, GFP and RFP fusions with the PTS1 and PTS2 localization signal were transformed into the wild type PH-1 and Δ*FgPex3* knock-out strains. The GFP and RFP transformants in the Δ*FgPex3* background showed a diffuse fluorescence pattern across the cytoplasm suggesting the absence of mature peroxisomes. The Δ*FgPex3* strain showed a minor, non-significant reduction in growth on various sugar carbon sources. In contrast, deletion of *FgPex3* affected fatty acid β-oxidation in *F. graminearum* and significantly reduced the utilization of fatty acids. Furthermore, the Δ*FgPex3* mutant was sensitive to osmotic stressors as well as to cell wall-damaging agents. Reactive oxygen species (ROS) levels in the mutant had increased significantly, which may be linked to the reduced longevity of cultured strains. The mutant also showed reduced production of conidiospores, while sexual reproduction was completely impaired. The pathogenicity of Δ*FgPex3*, especially during the process of systemic infection, was strongly reduced on both tomato and on wheat, while to production of deoxynivalenol (DON), an important factor for virulence, appeared to be unaffected.

## Introduction

Peroxisomes are a class of dynamic single-membrane-bound organelles, found in almost all eukaryotic cells. In recent years, it has become increasingly clear that peroxisomes are involved in a wide range of important cellular functions, including long-chain fatty acid β-oxidation, scavenging of reactive oxygen species (ROS), glyoxylate detoxification, and generation of reactive nitrogen species (RNS) signal molecules (Wanders and Waterham, 2006; Brown and Baker, 2008; Sandalio and Romero-Puertas, 2015; Tripathi and Walker, 2016). In addition, peroxisomes display multiple functions in filamentous fungi, such as the formation of Woronin bodies, which can seal septal pores after cellular wounding (Wanner and Theimer, 1982), metabolism of specific carbon sources (Hynes et al., 2006), and the biosynthesis of secondary metabolites (Brakhage et al., 2004; Meijer et al., 2010; Reverberi et al., 2012). Moreover, they are involved in virulence (Min et al., 2012) and sexual development (Peraza-Reyes and Berteaux-Lecellier, 2013).

The biogenesis of peroxisomes requires multiple peroxisomal proteins encoded by PEX genes (Distel et al., 1996). Currently, more than 30 PEX genes have been implicated in for instance the import of peroxisomal matrix proteins, assembly of the peroxisomal membrane, peroxisome proliferation and maintenance (Wanders and Waterham, 2005; Sibirny, 2016). All peroxisome matrix proteins are synthesized in the cytosol and transported to the peroxisome by targeting. There are two distinct peroxisomal targeting signals: PTS1 or PTS2 (Lazarow and Fujiki, 1985). PTS1 is located at the C-terminal end of matrix proteins and consists of a highly conserved tripeptide of -[S/C/A]-[K/R/H]-[L/M] (Notzel et al., 2016). PTS2 contains a conserved sequence [R/K]-[L/V/I]-X5-[H/Q]-[L/A], located at the N-terminal of matrix protein (Gould et al., 1989; Petriv et al., 2004). PEX5 and PEX7 are receptors for PTS1 and PTS2, respectively, that escort the cargo to the peroxisome membrane (Leon et al., 2006). In addition, PEX3, PEX16 and PEX19 are essential for the proper localization of peroxisome membrane proteins (PMPs). PEX3 has been proposed to act as a docking factor for PEX19 and to be involved in peroxisomal membrane biogenesis. In the early stages of peroxisome formation, PEX19 binds PMPs in the cytosol through a peroxisomal membrane-targeting signal (mPTS), composed of a PMP-binding domain and a membrane-anchoring domain (Rottensteiner et al., 2004; Matsuzono et al., 2006; Schueller et al., 2010). PEX3 can form a ternary complex with PEX19 and PMPs. The binding of PEX3 and PEX19 is an essential step in the targeting of PMPs to the peroxisomal membrane and absence of either PEX3 or PEX19 results in peroxisome deficiency (Pieuchot and Jedd, 2012).

Fusarium head blight, caused by the filamentous ascomycete *F. graminearum* species complex (FGSC), is one of the most devastating diseases affecting global wheat and barley production (Goswami and Kistler, 2004). *F. graminearum* is the predominant species and occurs in most FHB areas around the world (Bai and Shaner, 2004; Zhang et al., 2012; van der Lee et al., 2015). In recent years, FHB has attracted attention not only because of yield losses of economic importance, but also because of the resulting mycotoxin contamination, e.g., deoxynivalenol and zearalenone, which present a threat to the safety of human and livestock (Windels, 2000; Goswami and Kistler, 2004). *F. graminearum* produces both sexual (ascospores) and asexual (macroconidia) propagules, but ascospores are generally considered to be the primary inoculum of the disease (Bai and Shaner, 1996). These ascospores are formed in sexual fruiting bodies called perithecia and are forcibly discharged into the air to facilitate dispersal. The period of flowering is the most susceptible stage of wheat for head blight, and the presence of ascospores during this period is a critical epidemiological factor.

Few reports on the role of peroxisomes on growth, development and toxin production are available for *F. graminearum*. Deletion of *FgPex5* and *FgPex6* genes resulted in decreased metabolism of ROS, retarded vegetative growth on media with long-chain fatty acids, reduced sexual development and diminished pathogenicity (Min et al., 2012). A recent study (Chen et al., 2018) showed that peroxisomal proliferation was highly stimulated upon induction of DON biosynthesis and during plant infection by *F. graminearum*. Furthermore, deletion of *FgPex13*, *FgPex14* or *FgPex33* exhibited an increased accumulation of endogenous ROS, reduced phosphorylation of MAP kinase *FgMgv1* and reduced virulence (Chen et al., 2018). *FgPex4*, *FgPex1* and *FgPex10* are involved in cell wall integrity (Zhang et al., 2019a, b). These results demonstrate that peroxisomes play pivotal roles in development, cell wall integrity, DON biosynthesis and virulence in *F. graminearum*.

In this study, we investigated the function of PEX3 in *F. graminearum*, as there are no reports on the role of PEX3 on the peroxisome assembly in *F. graminearum* or any other filamentous fungi. Our results demonstrate that PEX3 is crucial for the formation of mature peroxisomes, hyphal development, conidiation, sexual reproduction, the utilization of long-chain carbon sources, ROS metabolism, stress responses, and pathogenicity in *F. graminearum*.

## Materials and Methods

### Strains and Cultural Conditions

*Fusarium graminearum* wild-type PH-1 and transformants were cultured on PDA (200 g potato, 20 g dextrose, 20 g agar per liter) agar plates at 25^*o*^C. For growth assays, the growth rates on PDA, complete medium (CM) (1 g KH_2_PO4, 0.5 g MgSO_4_⋅7H_2_O, 0.5 g KCl, 2 g NaNO_3_, 200 μL of trace element solution (5 g citric acid, 5 g ZnSO_4_⋅7H_2_O, 0.25 g CuSO_4_⋅5H_2_O, 50 mg MnSO_4_⋅H_2_O, 50 mg H_3_BO_3_ and 50 mg NaMoO_4_⋅2H_2_O per 100 mL), 10 mL of vitamin stock solution (100 mg thiamine, 75 mg nicotinamide, 50 mg ascorbic acid, 5 mg folic acid, 5 mg biotin, 30 mg riboflavin, 75 mg pyridoxine, 200 mg Choline chloride, 5 mg p-aminobenzoic acid, 200 mg Ca-pantothenate, 4 g Inositol, absolute ethanol and ddH_2_O are volumetric to 1 L in a ratio of 1:1), 2 g yeast extract, 2 g peptone, 1 g casamino acids, 30 g sucrose and 20 g agar per liter) and minimal medium (MM) (1 g KH_2_PO4, 0.5 g MgSO_4_⋅7H_2_O, 0.5 g KCl, 2 g NaNO_3_, 200 μL of trace element solution, 30 g sucrose and 20 g agar per liter) were measured during 3 days after inoculation. Sensitivity to various stress agents was tested by growing the strains on MM supplemented with either 0.08 M LiCl, 1 M NaCl, 1 M Sorbitol, 1.2 M KCl, 1 M glucose. Cell wall integrity was evaluated by inoculating the strains on MM amended with 0.01% SDS, 5 mM caffeine, 0.3 g/L congo red (CR) or 0.3 mg/L calcofluor white. Determination of utilization of different carbon sources was conducted on MM supplemented with either 2% sucrose, 40 mM ethanol (C2), 40 mM sodium butyrate (C4), 40 mM sodium valerate (C5), 40 mM sodium caproate (C6), 3 mM sodium laurate (C12), 3 mM sodium tridecanoate (C13), 3 mM sodium myristate (C14), 3 mM sodium laurate (C16) or sodium oleate (C18) as the sole carbon source. Five replicates were used for each strain and three independent repeats of the experiment were performed.

### Strain Construction

For deletion of *FgPex3* gene, we firstly constructed a *FgPex3* gene knockout vector as described below. Genomic DNA of wild-type strain PH-1 was used as template to amplify upstream and downstream fragments of *FgPex3* using primer pairs R1/R2 and R3/R4, respectively followed by ligation into vector pKH-KO, carrying the hygromycin resistance gene *hph*. For the construction of the gene complementation vector, the fragment containing promoter, gene, and terminator of *FgPex3* was amplified using primers R5/R6 and ligated into vector pKN (carrying the G418 resistance gene *neo*). Furthermore, we constructed a vector named as pPTS (carries the G418 resistance gene *neo*), which contains a GFP-PTS1 and a PTS2-DsRed fusion gene. PTS1 (SKL) was fused to the C-terminus of GFP and PTS2 was added to the N-terminus of DsRed. The GFP ORF with PTS1 signal was amplified with the forward primer GFP-Nco1 and the reverse fusion primer GFP-SKL that contains a tripeptide SKL encoding serial. The DsRed ORF with PTS2 signal was amplified using the forward fusion primer DsRed-F, which contains a PTS2 encoding region derived from N-terminal of *Saccharomyces cerevisiae* thiolase gene (GeneID: 854646), and the reverse primer DsRed-Kpn1. This vector was used as peroxisomal marker. The gene deletion, complementation and PTS-containing constructs were transformed into corresponding strains using the protocols described previously (Turgeon et al., 1987). PDA plates containing 100 μg/mL hygromycin B (AMRESCO, United States) or 200 μg/mL G418 (AMRESCO, United States) were used to screen the corresponding transformants. Putative positive transformants were identified by PCR assays, R7/R8 and H850/H852 primers were used for detecting the present of *FgPex3* and *hph* gene respectively. Transformants were further confirmed by Southern hybridization, *FgPex3* and *hph* probe were amplified by R11/R12 and R13/R14. The primers used are listed in Supplementary Table S1.

### Conidiation Assays

For conidiation assays, three mycelial plugs (9 mm in diameter) of each strain were inoculated into mung bean liquid medium (MBL) (30 g mung bean, 1 L ddH_2_O, boiled for 10 min, filtered and sterilized) and incubated at 25°C on a shaking table (180 rpm). Each strain was set up in three technical replicates. After 4 days of cultivation, the number of conidia was counted for each strain using a hemocytometer. Conidial sizes were observed with a Leica TCS SP5 imaging system.

### Sexual Reproduction Assay

For sexual reproduction, mycelial plugs of each strain were placed onto carrot agar (400 g carrot, 20 g agar per liter) medium and cultured at 25°C. After 4 days, aerial hyphae were removed and the plates were incubated under a cycle of 12 h of UV-light and 12 h of darkness for more than 1 week, during which period aerial hyphae were pressed down with 0.2% Tween-60 whenever they appeared. Formation of the perithecia was observed under a digital microscope VHX-2000 (KEYENCE, United States).

### Fluorescence Microscopy

Localization of GFP- and DsRed proteins in spores and mycelium was detected using a Zeiss LSM800 Confocal System (Germany), with excitation at 488 nm and emission at 520 nm for GFP, or excitation at 543 nm and emission at 607 nm for DsRed.

### Measurement of ROS Accumulation and Viability

The wild-type strain PH-1 and the *FgPex3* gene deletion mutant were inoculated into CM medium or CM medium containing 0.6 M NaCl for 3 days. Then the strains were stained with 20 mL of 0.2% NBT and cultured at 25°C for 30–45 min. Finally, excess of staining agent was removed by rinsing with ethanol and plates were placed in the incubator for 30–45 min and then used for photo recording. Mycelial plugs from the original cultures were transferred to fresh CM agar plates were cultured for 7 days, 30 days, 60 days, 90 days, respectively. The growth was observed and photographed.

### Pathogenicity Determination

Conidia produced in the MBL medium were harvested and resuspended to a final concentration of 10^6^ conidia/mL in sterile water as previously described (Kong et al., 2018). For infection on flowering wheat (cv. Yangmai 158) grown in the greenhouse, a spikelet at the lower-middle part of a wheat head was inoculated with 10 μL of conidial suspension, with 30 replicates per strain. The inoculated ear was sprinkled with water and incubated in a humidity chamber for 3 days. Symptomatic spikelets in each inoculated wheat head were recorded and photographed. Infection assays on tomato were performed as previous described (Di Pietro et al., 2001). In brief, a 10 μL aliquot of a conidial suspension (10^6^ conidia/mL) was injected into wounded tomato after surface disinfection. Inoculated tomatoes were incubated in 25°C, 80–100% humidity with 12 h of daylight and were investigated 3 days after inoculation.

### DON Production Assays

One milliliter spore suspension with a concentration of 10^5^ spores/mL was added to a flask with 30 g of sterilized rice kernels. Each strain was cultured for 7 days with 3 replicates at 25°C, shaking the flasks on a daily basis. The infected rice medium was dried in an oven at 60°C and subsequently ground to a fine powder. 5 g samples were mixed with 25 mL distilled water for 3 min, centrifuged and filtered, and 50 μL filtrate was taken for analysis using the DON Plate Kit ELISA from Beacon Analytical Systems (Portland, ME, United States) according to the manufacturer’s instructions.

## Results

### Identification of *FgPex3* in *F. graminearum*

**FIGURE 1:**
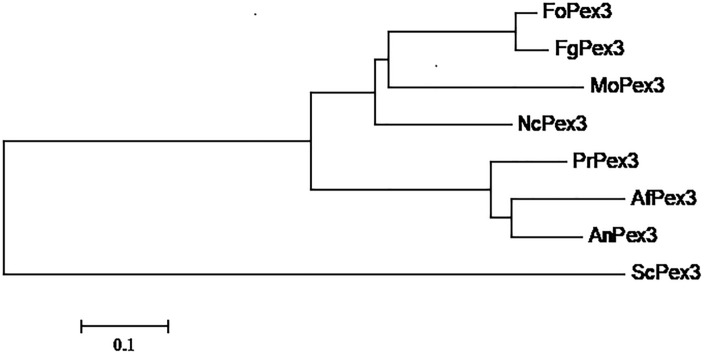
Phylogenetic relationship of Pex3 protein homologs. Phylogenetic relationship of Pex3 homologs calculated using the neighbor-joining method with the MEGA 6.0 program. Abbreviations and accession numbers of the sequences are as follows: FoPex3 (EWZ44752.1) from *Fusarium oxysporum* Fo47; FgPex3 (XP_011326612.1) from *Fusarium graminearum* PH-1; MoPex3 (XP_003717143.1) from *Magnaporthe oryzae* 70–15; NcPex3 (XP_962987.1) from *Neurospora crassa* OR74A; PrPex3 (XP_002567187.1) from *Penicillium rubens* Wisconsin 54–1255; AfPex3 (XP_002383001.1) from *Aspergillus flavus* NRRL3357; AnPex3 (XP_659885.1) from *Aspergillus nidulans* FGSC A4; ScPex3 (NP_010616.3) from *Saccharomyces cerevisiae* S288C.

The *F. graminearum* PEX3 ortholog (FGSG_06942, assigned as *FgPex3* in the present work) was identified from the genome data-base (King et al., 2015) through BLASTP searches using the *S. cerevisiae* PEX3 protein (GenBank accession number: NP_010616) as a query. The *FgPex3* gene consists of three exons and two introns, and encodes a polypeptide of 523 amino acid residues. The predicted protein sequences of *FgPex3* exhibited moderate identity (25%) with that of *S. cerevisiae*. The PEX3 amino acid sequences from several pathogenic fungi (*Magnaporthe oryzae*, *Neurospora crassa*, *Aspergillus flavus*, *A. nidulans* and *Fusarium oxysporum*) were downloaded from the NCBI database, and the phylogenetic tree based on the amino acid sequences revealed that *FgPex3* is most closely related to *FoPex3* and *MoPex3* (Figure 1).

### Construction of the Δ*FgPex3* Mutant and *FgPex3* Complemented Strain

**FIGURE 2:**
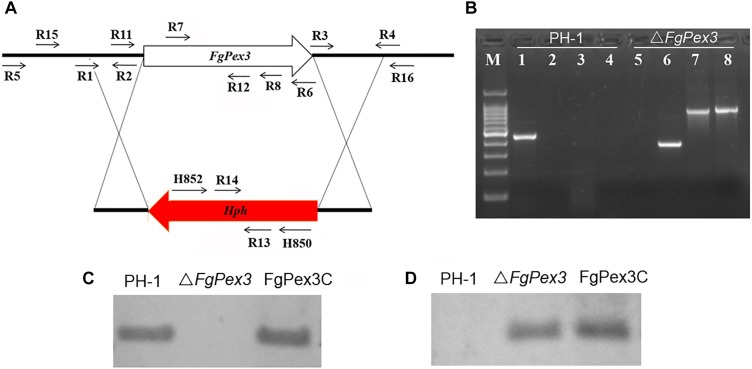
The *FgPex3* gene replacement construct. **(A)** Schematic representation gene disruption strategy. *FgPex3* is denoted by an open arrow; hygromycin resistance cassette (*hph*) is represented by a red arrow. The annealing sites of primers are indicated by small black arrows. **(B)** PCR analysis of PH-1 and *FgPex3* deletion mutant using primers R7/R8, H850/H852, H852/R16, and R15/H850, respectively. 1–4: PCR products amplified with the above primers using PH-1 as template; 5–8: PCR products amplified with the above primers using Δ*FgPex3* as template. **(C,D)** Southern blot hybridization analyses of transformants with hybridization probe R11/R12 (*FgPex3*) and R13/R14 (*hph*).

To determine the functions of *FgPex3*, we performed gene replacement through homologous recombination (Figure 2A), and the resulting hygromycin-resistant strains were tested by PCR. As expected, the fragment within the *FgPex3* nucleotide sequence could be detected in the wild-type, but not in the Δ*FgPex3* mutant (Figure 2B). Replacement of the *FgPex3* gene in these mutants was further confirmed by Southern hybridization (Figure 2C). Complementation of this mutant was obtained by ectopic insertion of the *FgPex3* gene into the genome and identification by PCR (primers R7/R8) (data not shown) as well as by Southern hybridization (Figure 2D).

### *FgPex3* Is Indispensable for the Mature Peroxisome Biogenesis

**FIGURE 3:**
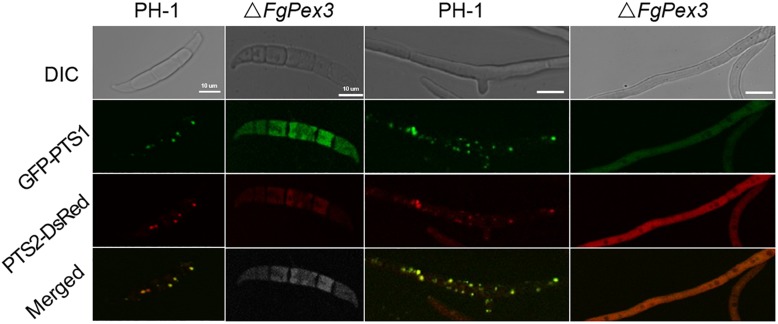
Peroxisomal targeting fusion proteins in spores (left) and mycelia (right) of PH-1 and Δ*FgPex3*. The fluorescence of GFP-PTS1 and PTS2-DsRed localized to peroxisomes as discernable structures in the wild type strain PH-1, whereas both types of fluorescence exhibited a diffuse pattern in the cytoplasm of the mutant Δ*FgPex3*. DIC, differential interference contrast; GFP-PTS1, green fluorescent protein contained in the peroxisomal targeting signal 1; DsRed-PTS2, red fluorescent protein contained in the peroxisomal targeting signal 2; Merge, overlays of green and red fluorescence proteins images. Scale bar = 10 μm.

It is reported that yeast *pex3 atg1* mutant cells contain abundant reticular and vesicular structures — preperoxisomal vesicles (PPVs) that harbor key proteins of the peroxisomal receptor docking complex Pex13 and Pex14 as well as the matrix proteins Pex8 and alcohol oxidase. Besides, Pex3 first appeared on the PPVs upon its reintroduction in *pex3* cells, after which these structures mature into normal peroxisomes (Aksit and van der Klei, 2018). That is to say, Pex3 is essential for the maturation of peroxisomes. To test whether the deletion of *FgPex3* had any effect on the peroxisome biogenesis, we analyzed the localization of the fluorescent hybrid proteins GFP-PTS1 and PTS2-DsRed, which represent the distribution of peroxisomes. In conidia and in mycelium of wild-type strain PH-1, the two marker proteins were found to be well distributed in peroxisomes (visible as discrete entities in Figure 3), while in spores and mycelia of Δ*FgPex3*, the two marker proteins exhibited a diffuse fluorescence pattern across the cytoplasm (Figure 3). This indicated that *FgPex3* is required for the biogenesis and formation of mature peroxisomes.

### *FgPex3* Influences Vegetative Growth

**FIGURE 4:**
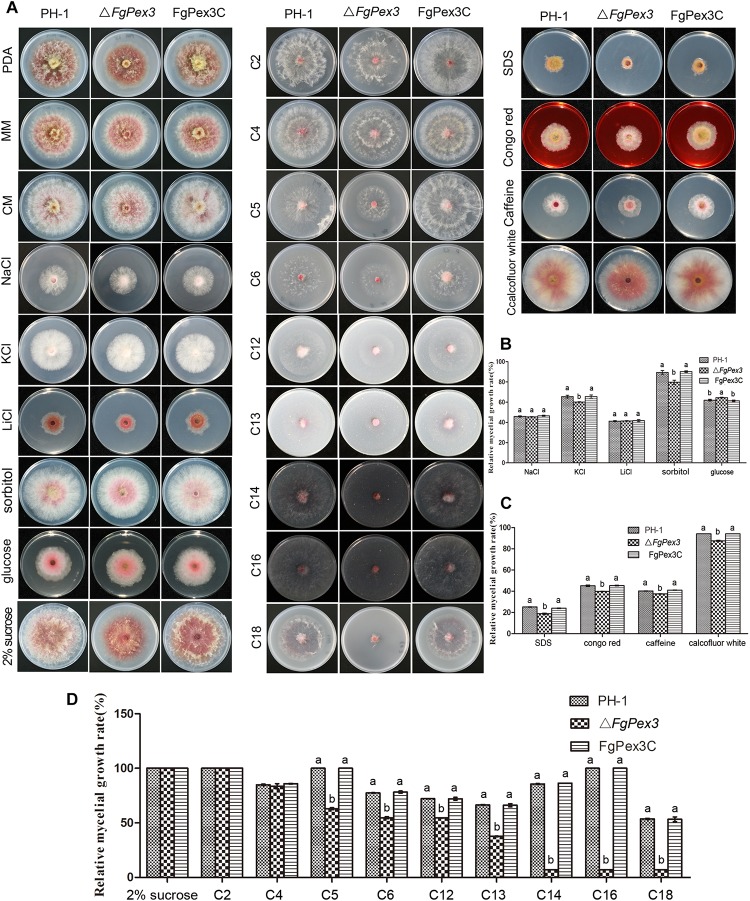
Growth analysis of *F. graminearum* strains under different culture conditions. **(A)** The wild-type PH-1, deletion mutant and the complemented strain were cultured on three basic media (PDA, CM, and MM) as well as MM plates supplemented with osmotic stress agents (NaCl, KCl, LiCl, sorbitol or glucose), MM plates amended with either cell wall damaging agents (SDS, Congo red, caffeine or calcofluor white) or with various carbon sources (2% sucrose, 40 mM sodium acetate (C2), 40 mM sodium butyrate (C4), 40 mM sodium valerate (C5), 40 mM sodium hexanoate (C6), 3 mM laurate acid (C12), 3 mM tridecanoic acid (C13), 3 mM myristic acid (C14), 3 mM palmitic acid (C16) or 3 mM oleic acid (C18) as the sole carbon source) at 25°C for 3 days. **(B–D)** Colony diameters of the tested strains were measured to calculate the inhibition rate and statistically analyzed. Error bars represent the deviation from five replicates, and the same letter on the bars for each treatment indicates no significant difference at *P* = 0.05.

As shown in Figure 4A, the colony morphology of Δ*FgPex3* mutant strains had great differences on PDA medium compared with that of wild-type PH-1 and the complemented strain, FgPex3C. On PDA medium, Δ*FgPex3* showed short and spare aerial hyphae, while on MM and CM media, no morphological differences could be observed between the three strains, with the exception that Δ*FgPex3* seemed to grow slightly slower. The colony diameter of Δ*FgPex3* (43.36 + 2.21 mm) was significantly smaller than that of PH-1 (49.68 + 2.12 mm) and FgPex3C (49.95 + 3.21 mm) after 3 days at 25°C (*P* < 0.05).

### *FgPex3* Is Essential for Stress Responses and Cell Wall Integrity of *F. graminearum*

To determine whether Δ*FgPex3* was defective in various forms of stress response, we assayed its growth on MM plates with different stress agents compared to growth on MM plates without any stress agent. In osmotic sensitivity tests, Δ*FgPex3* proved more sensitive to potassium chloride (1.2 M) and sorbitol (1 M), and its relative growth rates decreased significantly (*P* < 0.05). When growing on a medium containing glucose (1 M), the sensitivity of Δ*FgPex3* decreased and the relative growth rates increased (*P* < 0.05). Δ*FgPex3* did not show any obvious response to sodium chloride (1.2 M) or lithium chloride (0.1 M) (Figures 4A,B). To investigate whether *FgPex3* plays a role in maintaining cell wall integrity in *F. graminearum*, we examined how the gene deletion mutant responded to a range of cell wall-damaging agents. As shown in Figure 4A, the Δ*FgPex3* mutant was more sensitive toward SDS (0.01%), Congo red (0.3 mg/L), caffeine (5 mM) and calcofluor white (0.3 mg/L) than either the wild-type or the complemented strain and its growth rate was significantly decreased (Figure 4C, *P* < 0.05).

### Deletion of *FgPex3* Affects the Capacity to Utilize of Long-Chain Carbon Sources

For the determination of the capacity to use carbon sources, PH-1, Δ*FgPex3* mutant and FgPex3C was cultured on MM plates with 2% (58.4 mM) sucrose, 40 mM sodium acetate (C2), 40 mM sodium butyrate (C4), sodium valerate (C5), 40 mM sodium caproate (C6), 3 mM sodium laurate (C12), 3 mM sodium tridecanoate (C13), 3 mM sodium myristate (C14), 3 mM sodium palmitate (C16) or 3 mM sodium oleate (C18) as the sole carbon source.

Compared with wild-type strain PH-1 and the complemented strain FgPex3C, Δ*FgPex3* mutant showed no significant difference in relative growth rates on MM medium with sucrose, C2 and C4 as the sole carbon sources. In contrast, relative growth rates of Δ*FgPex3* mutant on MM medium with C5, C6, C12, C13, C14, C16 and C18 as sole carbon sources were significantly decreased (*P* < 0.05). With the increase of fatty acid carbon chain length, the growth of the Δ*FgPex3* mutant was reduced to very tiny colonies on C14, C16 or C18 media (Figures 4A,D).

### *FgPex3* Is Involved in the Conidial Production and Sexual Development

**FIGURE 5:**
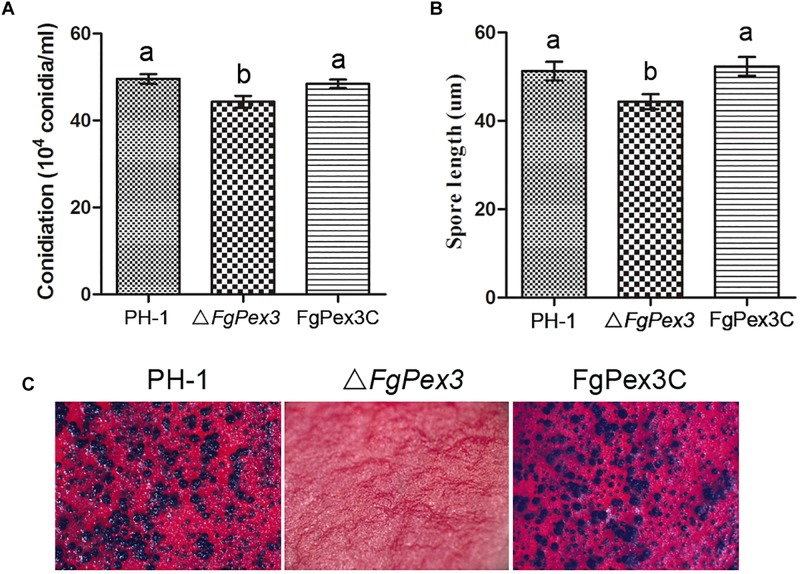
Conidiation and sexual reproduction of PH-1, Δ*FgPex3* and FgPex3C. **(A)** Number of conidia produced in MBL medium during 6 days at 25°C. Means and standard errors were calculated from three independent replicates. Error bars represent standard deviation. Values on the bars followed by the same letter indicate no significant difference at *P* = 0.05. **(B)** Size of conidia produced by PH-1, ΔFgPex3 and FgPex3C strains. Error bars indicate the standard deviations of three repeated experiments. Values on the bars followed by the same letter are not significantly different at *P* = 0.05. **(C)** Perithecia formation of PH-1, Δ*FgPex3* and FgPex3C on carrot agar plates were photographed at 2 weeks post-fertilization. Mutant Δ*FgPex3* failed to produce any perithecia.

The Δ*FgPex3* mutant produced significantly less conidia compared to the wild-type and complemented strain FgPex3C in MBL medium (*P* < 0.05) (Figure 5A). Furthermore, microscopic examination showed that the conidia of Δ*FgPex3* mutant were shorter than those of wild-type and complemented strains (Figure 5B). These results indicate that *FgPex3* is important for conidiogenesis.

Because ascospores play a vital role in the primary infection of *F. graminearum*, the production of perithecia on carrot agar plates was determined as described (Kong et al., 2018). At 14 days post fertilization, the wild type strain PH-1 and the complemented strain FgPex3C produced mature perithecia, while the Δ*FgPex3* mutant failed to produce any perithecia under the same conditions (Figure 5C). The absence of perithecia indicates that *FgPex3* is essential for sexual reproduction.

### *FgPex3* Affects the Virulence of *F. graminearum*

**FIGURE 6:**
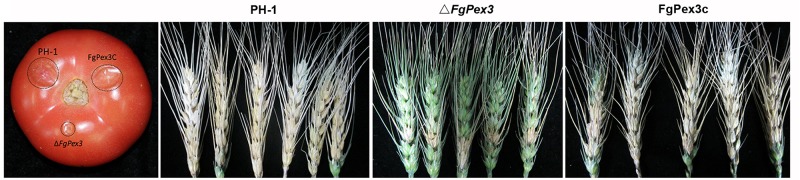
Virulence of PH-1, Δ*FgPex3* and FgPex3C on infected plants. Virulence of the three strains was determined by point inoculations of conidial suspensions on five tomato fruits and 30 wheat heads. Infected tomato fruits and wheat heads were photographed at 3 days and 21 days post inoculation, respectively.

**FIGURE 7:**
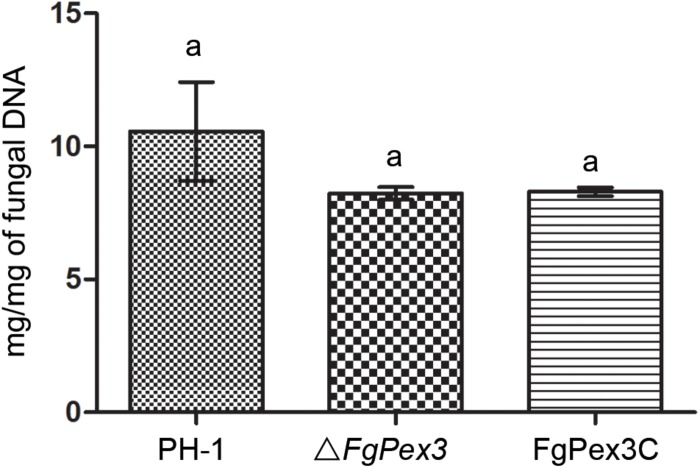
DON production of the wild-type, mutant and complemented strain on infected rice kernels. Values on the bars followed by the same letter indicate no significant difference at *P* = 0.05.

Infection experiments were performed to see if the Δ*FgPex3* mutant was affected in the pathogenicity on tomato and wheat. Inoculations were performed on tomatoes and wheat spikelets. The infected area of the Δ*FgPex3* mutant on tomato was substantially smaller and did not expand beyond the inoculation site (Figure 6). Most of the wheat heads inoculated with the Δ*FgPex3* mutant showed rarely symptoms. Under the same experimental conditions, PH-1 and FgPex3C strains caused extensive lesions on tomato fruits and typical scab symptoms on wheat heads (Figure 6). These results revealed that *FgPex3* plays an important role in the infection of (host) plants. The displayed reduced virulence and failure to spread to neighboring spikelets is reminiscent of mutants in the DON pathway (Proctor et al., 1995; Seong et al., 2009). However, when we examined the DON accumulation in the *FgPex3* deletion mutant, no significant difference in DON production among the wild type, Δ*FgPex3* mutant and the complemented strain FgPex3C was observed (Figure 7). This indicates that DON production is still possible and does not require *FgPex3*.

### ROS Accumulation and Viability of Δ*FgPex3*

**FIGURE 8:**
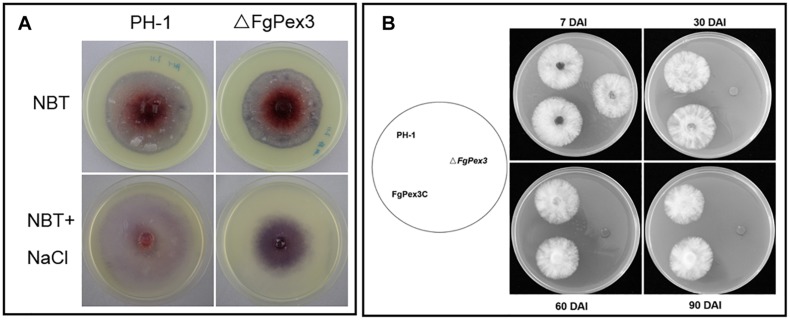
Reduced ability of Δ*FgPex3* to scavenge reactive oxygen species (ROS) survival assay. **(A)** Tetranitroblue tetrazolium chloride (NBT) staining for ROS accumulation in cultures of Δ*FgPex3* and the wild-type. In plates at the bottom 0.6 M NaCl was added to promote ROS production. The dark blue color of the colony indicated ROS accumulation. **(B)** Strains PH-1,Δ*FgPex3*, FgPex3C were inoculated onto CM plates and incubated for 7–90 days. Mycelial plugs from the cultures were transferred to fresh CM agar plates, and the viability of the culture was observed. DAI, days after incubation.

Under different stress conditions, most eukaryotic organisms will produce large amounts of ROS, including hydrogen peroxide (H_2_O_2_), superoxide radicals and singlet oxygen (Baxter et al., 2014; Mor et al., 2014), to resist environmental changes. Nitroblue tetrazolium (NBT) can react with superoxide radicals to form blue formazan. When NBT was added to the growth plate, the color of the plate on which Δ*FgPex3* mutant was growing on, was significantly deeper than that of wild-type strain PH-1 (Figure 8A), indicating increased accumulation of ROS in Δ*FgPex3*.

To investigate a possible function of *FgPex3* on the viability of *F. graminearum*, we performed survival assays of mycelia. Δ*FgPex3* mutant, wild-type strain PH-1 and FgPex3C were cultured for 7, 30, 60, and 90 days in CM medium, and mycelial plugs were inoculated onto fresh CM plates. There was no significant difference in viability among 7-day-old mycelia. However, 30-, 60- and 90-day-old mycelia of Δ*FgPex3* were no longer viable. Both PH-1 and FgPex3C strains remained viable (Figure 8B). In conclusion, the deletion of *FgPex3* may affect the metabolism of peroxisomes in *F. graminearum*, and the accumulation of harmful substances such as ROS causes cell death, resulting in a decline of viability of Δ*FgPex3* beyond seven days of cultivation.

## Discussion

Peroxisomes are involved in a variety of metabolic reactions in almost all eukaryotic cells. Previously, several peroxisomal proteins were shown to be associated with biosynthesis of secondary metabolites and virulence in filamentous fungi (van der Klei and Veenhuis, 2013). However, the role of PEX3, an integral peroxisomal membrane protein that interacts with the cytosolic shuttling receptor PEX19 was not studied. This is surprising, as PEX3 was suggested to be responsible for the early steps of peroxisome biogenesis as well as assembly of peroxisomal membranes and/or the correct translocation of PMPs (Ghaedi et al., 2000; Schliebs and Kunau, 2004). Here, we functionally analyzed PEX3 from *F. graminearum* using targeted gene deletion methods, and found that the loss of *FgPex3* led to a plethora of developmental defects in hyphal growth, asexual and sexual reproduction, stress responses as well as virulence in *F. graminearum*. This is the first demonstration that PEX3 plays an important role in pathogenicity of filamentous fungus.

Eukaryotic cells are highly organized and specific functions are performed by specialized cellular compartments such as Golgi apparatus, ER, vacuole, mitochondria nucleolus and peroxisomes. Peroxisomes are devoid of genome or transcription machinery, therefore, all peroxisomal membrane and matrix proteins have to be imported from the cytosol (Purdue and Lazarow, 2001). It has been suggested that all PMPs accumulate in the endoplasmic reticulum (ER) in *pex3* cells, and that upon reintroduction of Pex3 these PMPs are incorporated in two types of vesicles that fuse to form mature peroxisomes (van der Zand et al., 2010, 2012). According to this model, Pex3 is important for the exit of PMPs from the ER into PPVs. However, recent findings showed that *pex3 atg1* cells contain PPVs, which are the targets for the reintroduced Pex3, which then mature into normal peroxisomes (Knoops et al., 2014; Wroblewska et al., 2017). Based on this, Pex3 is required for the formation of mature peroxisomes. In our study, Δ*FgPex3* matrix proteins (PTS1 or PTS2 containing proteins) were mislocated, demonstrating that *FgPex3* is also required for the formation of mature peroxisomes in *F. graminearum*.

Fatty acid β-oxidation is the main pathway for fatty acid degradation in eukaryotes. In plants, β-oxidation pathway plays a central role in plant reproduction, seed development and germination and post-germinative seedling establishment (Pan et al., 2018). Earlier studies in yeast suggest that fungi lack the mitochondrial fatty acid β-oxidation pathway (Hiltunen et al., 1992; Kurihara et al., 1992). However, related studies have shown that *A. nidulans* has both mitochondrial and peroxisomal fatty acid β-oxidation pathways (Maggio-Hall and Keller, 2004). Bioinformatic analyses indicate that both these pathways exist in most filamentous fungi, including *F. graminearum* (Shen and Burger, 2009). It has been reported that deletion of *pex* genes in various fungi can cause defects in fatty acid utilization. For example, deletion of the *pex7* gene in *M. oryzae* and in *F. graminearum* leads to short-chain fatty acid utilization disorder, while gene deletion of *pex5* or *pex6* in *F. graminearum* gave rise to reduced growth rate on media containing either long-chain or short-chain fatty acids (Goh et al., 2011; Min et al., 2012). Our study showed that the deletion of *FgPex3* in *F. graminearum* significantly reduced the utilization of both long-chain (*C* > = 12)and short-chain (*C* = 5 or 6) fatty acids. However, unlike the Δ*FgPex5* and Δ*FgPex6* deletion mutants, deletion of *FgPex3* gradually decreased the utilization of fatty acids with increasing of carbon chain length and it was almost impossible to grow and expand on medium with carbon chains longer than 14 (myristic acid, palmitic acid, oleic acid). In studies with animal cells it was also observed that the substrate for peroxisomal fatty acid β-oxidation is primarily long-chain to very-long-chain fatty acids (Reddy and Hashimoto, 2001). We hypothesize that similarly to these animal systems the deletion of the *FgPex3* affects the fatty acid β-oxidation process in *F. graminearum*, and significantly reduces the utilization of long-chain fatty acids.

Triacylglycerol produced by fatty acid β-oxidation is important for the production of ascospores by Ascomycetes (Lee et al., 2011). During the process of sexual reproduction of ascomycetes, the expression of genes associated with peroxisomal proteins and fatty acids biosynthesis is up-regulated and lipid content is gradually reduced (Guenther et al., 2009). Effective use of lipids can provide both energy and metabolites required for formation female sexual reproductive structures. In *Podospora anserina* and *A. nidulans*, peroxisomal proteins play important roles in sexual reproduction (Bonnet et al., 2006; Hynes et al., 2008). The *FgPex3* gene-deficient *F. graminearum* mutant was unable to produce perithecia on carrot agar medium, which could be linked to fatty acid β-oxidation in peroxisomes as these play an important role in sexual reproduction of *F. graminearum* (Peraza-Reyes and Berteaux-Lecellier, 2013).

Oxidative reactions are subject of the peroxisome metabolism, which generate massive amounts of ROS, mainly H_2_O_2_ (Chen et al., 2016). Besides H_2_O_2_, it has been demonstrated that peroxisomes also produce superoxide radicals as a consequence of their normal metabolism (Del Rio and Lopez-Huertas, 2016). ROS can be scavenged by catalases and peroxidases, which are abundant in peroxisomes. In *Hansenula polymorpha* the degradation of the peroxisomes caused by the exposure to high methanol conditions also resulted in an increase in formation of ROS (van Zutphen et al., 2011). In this study, the disruption of *FgPex3* in *F. graminearum* blocked peroxisome biogenesis and resulted in a significant increase in the accumulation of superoxide radicals, which reflects a higher ROS level. Like *FgPex5* and *FgPex6* deletion mutant, the survival of the *FgPex3* gene deletion mutant was significantly reduced. These results indicate that *FgPex3* is involved in mature peroxisomes formation, causing abnormal ROS metabolism, accumulation of ROS, and ultimately cell death.

The filamentous fungi, as an important group of plant pathogenic fungi, have attracted extensive attention for their pathogenicity. In the rice blast fungus, *M. oryzae*, the loss of MoPex5, MoPex7 or MoPex14/17 led to decreased conidia production, reduction of fatty acid utilization, ROS degradation and cell wall integrity as well as diminished virulence (Goh et al., 2011; Wang et al., 2013; Li et al., 2017). In *F. graminearum*, PEX5 and PEX6 deletion mutants showed greatly reduced pathogenicity (Min et al., 2012). In this study, we observed that the pathogenicity of the Δ*FgPex3* mutant is also strongly reduced both on its natural host wheat as well as on the alternative host tomato. To our knowledge, this is the first time that the fungal PEX3 has been found to be involved in pathogenicity.

Peroxisomal fatty acid β-oxidation plays an important role in the synthesis of glycerol and melanin, thereby affecting the infection of plant-pathogenic fungi like *Colletotrichum lagenarium* and *M. oryzae* in host plants (Kimura et al., 2001; Ramos-Pamplona and Naqvi, 2006; Wang et al., 2013). The *FgPex3* gene deletion mutant strain can produce lesions on tomato and infect inoculated wheat spikelets, which indicates that peroxisomes have little effects on the initial stage of pathogen infection. However, PEX3 plays a pivotal role during the process of systemic infection. Loss of peroxisomal function may result in reduced utilization of carbon sources from both the pathogen itself and from host plants, resulting in a slower growth rates. This slower growth rate could provide host plants time needed to prepare to ward off the fungal invasion, for instance by thickening the cell walls of the wheat head (Jansen et al., 2005), resulting in decreased pathogenicity. DON is an important factor for the virulence of *F. graminearum* on wheat. Although the disruption of *FgPex3* in *F. graminearum* still allowed DON production, pathogenicity is markedly reduced, indicating the importance of additional factors for virulence in Δ *FgPex3*.

In summary, we have functionally characterized *FgPex3*, an integral peroxisomal membrane protein involved in the mature peroxisomes biogenesis, fungal development and pathogenicity in *F. graminearum*. We discovered that *FgPex3* plays an essential role in the peroxisome biogenesis, fungal development, lipid degradation, scavenging of ROS, cell wall integrity and virulence. We also observed that the development of sexual fruiting bodies is blocked in the mutant. As the sexual ascospores are a critical driver for the epidemiology, fungicides that target the PEX3 or other proteins that are essential for peroxisome formation could be good targets for fungicide development.

## Data Availability

All datasets generated for this study are included in the manuscript and/or the Supplementary Files.

## Author Contributions

JF, WC, and HZ conceived and designed the study. XK and XW carried out the experiments, analyzed the data, prepared the figures and tables, and wrote the manuscript. JinX and JingX provided assistance for data analysis. HZ, BB, AD, TL, and CW wrote and revised the drafts of the manuscript. All authors read and approved the final manuscript.

## Conflict of Interest Statement

The authors declare that the research was conducted in the absence of any commercial or financial relationships that could be construed as a potential conflict of interest.
